# Dataset of chloroplast DNA sequences, *ndhC–trnV, rpoA, trnK–matK,* and *trnK–rps16* regions of *Platycodon grandiflorus* (Jacq.) A.DC. from Vietnam

**DOI:** 10.1016/j.dib.2026.112627

**Published:** 2026-02-25

**Authors:** Dung Manh Ngo, Luong Trong Vu, Hung Duc Nguyen, Thuy Thi Thu Vu, Mau Hoang Chu

**Affiliations:** Thai Nguyen University of Education, Thainguyen City 24000, Vietnam

**Keywords:** DNA marker, *Platycodon grandiflorous*, Nucleotide diversity, Phylogeny, Chloroplast DNA

## Abstract

*Platycodon grandiflorus* (Jacq.) A.DC., a member of the Campanulaceae family, is a well-known medicinal herb traditionally used to relieve coughs, promote expectoration, reduce inflammation, and protect the respiratory system. In recent years, numerous commercial products containing *Platycodon* extracts have been introduced to the market. However, during processing and distribution, the morphological and anatomical features of the raw material are often lost or altered, increasing the risk of misidentification or adulteration with morphologically similar species, thereby compromising product quality and therapeutic efficacy. Therefore, the application of DNA barcode markers is essential for accurate species identification and quality control of raw materials. This study presents a dataset of *P. grandiflorus* samples collected from natural populations and analyzes four chloroplast DNA regions (*ndhC-trnV, rpoA, trnK-matK*, and *trnK-rps16*) to support species identification. Phylogenetic analysis based on these sequences revealed that *P. grandiflorus* is closely related to, and clusters within, the Campanulaceae family, with high bootstrap support values (96–100%). These four chloroplast regions are proposed as potential DNA barcodes for the reliable identification and differentiation of *P. grandiflorus* from related species.

Specifications Table

**Table:** 

Subject	Biological Sciences
Specific subject area	Biotechnology, Genetics diversity, Molecular Phylogenetics, Evolution
Type of data	Raw, sequence data, tables, figures, text files
Data collection	Nucleotide sequence data of DNA regions were sequenced using an ABI 3130xL 16 capillary sequencer.
Data source location	Department of Genetics & Biotechnology, Faculty of Biology, Thai Nguyen University of Education, Vietnam
Data accessibility	Repository name: Mendeley Data Data identification number: 10.17632/p6kwdkkj3v.1 Direct URL to data: https://data.mendeley.com/datasets/p6kwdkkj3v/1

## 1 Value of the Data

• *Platycodon grandiflorus* (Jacq.) A.DC., belonging to the Campanulaceae family, is a traditional medicinal herb widely used to treat coughs, promote expectoration, alleviate inflammation, and support respiratory health. It contains diverse bioactive compounds with notable therapeutic potential.
• The dataset includes intergenic spacer sequences from four chloroplast DNA regions (*ndhC–trnV, rpoA, trnK–matK*, and *trnK–rps16*), analyzed to develop reliable molecular markers for precise species identification, particularly useful when plant materials are processed or in powdered form.
• The primer pairs Pla-ndhCV-F/Pla-ndhCV-R, Pla-rpoA-F/Pla-rpoA-R, Pla-trnK-matK-F/Pla-trnK-matK-R, and Pla-trnK-rps16-F/Pla-trnK-rps16-R effectively amplify chloroplast regions, *ndhC–trnV, rpoA, trnK–matK*, and *trnK–rps16*. It can be applied to species within the *Strobilanthes* genus (Acanthaceae) and other higher plants.
• Phylogenetic and evolutionary analyses based on these intergenic spacer regions provide valuable information on genetic relationships and evolutionary divergence within the *Platycodon* genus.

## 2 Background

*Platycodon grandiflorus* (Jacq.) A.DC., a member of the Campanulaceae family, is a traditional medicinal herb widely used in Vietnamese medicine to treat respiratory disorders [1]. Precise identification of this species is crucial to ensure the efficacy and safety of its therapeutic use.

Chloroplast DNA (cpDNA) is a circular, double-stranded molecule containing multiple gene regions that vary in their degree of conservation and sequence divergence [2]. Variations in nucleotide composition among these regions reflect genetic relationships or differentiation among plant taxa. Therefore, highly variable cpDNA regions are often utilized in phylogenetic analyses and serve as valuable molecular markers (DNA barcodes) for accurate species identification.

The complete chloroplast genome of *P. grandiflorus* has been sequenced and is available in GenBank under accession number NC_035624.1 [3]. To clarify phylogenetic placement and support reliable species identification, three intergenic spacer regions (*ndhC–trnV, trnK–matK, trnK–rps16*) and the *rpoA* gene of *P. grandiflorus* were analyzed.

## 3 Data Description

**Fig. 1:**
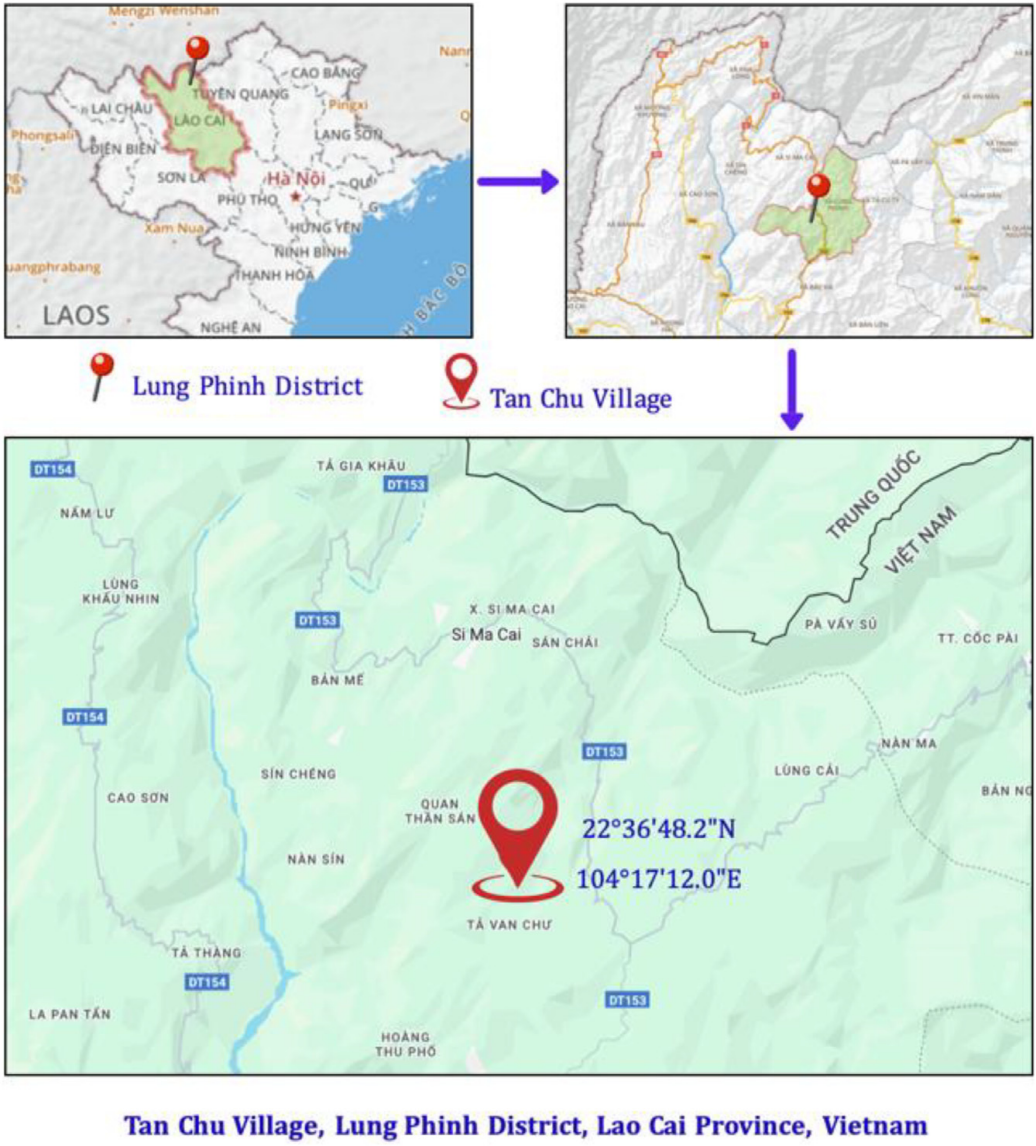
Map showing the sampling location of *Platycodon grandiflorum* (Jacq.) A.DC. in Tan Chu village, Lung Phinh commune, Lao Cai province, Vietnam (22°36′48.2″N 104°17′12.0″E).

*Platycodon grandiflorus* (Jacq.) A.DC., belonging to the Campanulaceae family, is a familiar medicinal plant with effects such as cough treatment, expectorant action, anti-inflammatory properties, and protection of the respiratory system. In this study, samples were collected in Tan Chu village, Lung Phinh commune, Lao Cai province, Vietnam (22°36′48.2″N 104°17′12.0″ E) (Fig. 1).

**Fig. 2:**
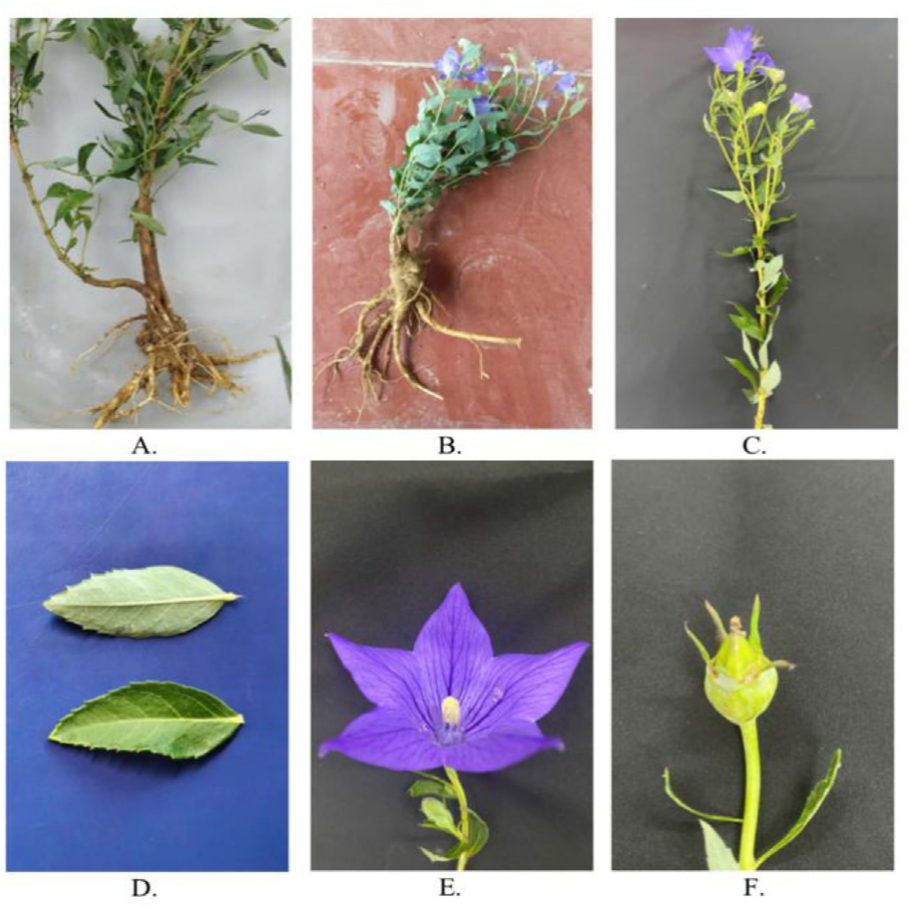
Morphological characteristics of *Platycodon grandiflorus* (Jacq.) A.DC was collected from Tan Chu village, Lung Phinh district, Lao Cai province, Viet Nam (22°36′48.2″N 104°17′12.0″E). A. Whole plant; B. Flowering plant; C. Flowering branch; D. Upper and lower surfaces of the leaf; E. Flower; F. Fruit.

*P. grandiflorus* is a perennial herb reaching 45–90 cm in height, with smooth and sparsely branched stems. Its cylindrical roots, 5–25 cm long, range in color from light yellow to brown and display prominent longitudinal wrinkles. Leaves are opposite or alternate, with serrated margins, dark green adaxial surfaces, and a whitish powdery coating on the abaxial side. The plant produces solitary, bell-shaped violet flowers with a five-lobed green calyx and develops inverted-ovoid capsules containing numerous small brown seeds (Fig. 2).

The complete chloroplast genome of *P. grandiflorus* (171,818 bp) has been sequenced and deposited in GenBank under accession number NC_035624.1 [3]. Nucleotide diversity (Pi) analysis identified three intergenic spacer regions, *ndhC–trnV, trnK–matK*, and *trnK–rps16,* and the *rpoA* gene as suitable targets for PCR primer design (Table 1). These regions were successfully amplified using the primer pairs Pla-ndhCV-F/Pla-ndhCV-R, Pla-rpoA-F/Pla-rpoA-R, Pla-trnK-matK-F/Pla-trnK-matK-R, and Pla-trnK-rps16-F/Pla-trnK-rps16-R. Sequencing results confirmed fragment lengths of 829 bp (*ndhC–trnV*), 993 bp (*rpoA*), 751 bp (*trnK–matK*), and 690 bp (*trnK–rps16*). The chloroplast DNA sequences (*ndhC–trnV, rpoA, trnK–matK*, and *trnK–rps16*) have been submitted to GenBank and were assigned access numbers by GenBank as follows: PX584285, PX584287, PX584286, and PX584284. The nucleotide sequences of *trnE-trnT, psbK-psbI, ycf1*, and *matK* are available on Mendeley Data [4] and they are presented in Table 2.

**Table 1:** Primer sequences were designed and used to amplify the *ndhC-trnV, rpoA, trnK-matK* and *trnK-rps16* regions of *Platycodon grandiflorus.*

No.	Primers name	Primer sequences (5′- 3′)	Product size (bp)	Ta (°C)
1	*Pla-ndhCV-F*	TAACTTTTTAGTTTAAGCAAGGA	817	43
	*Pla-ndhCV-R*	AGAGCTCTATAAATGCAAAAATAC		
2	*Pla-rpoA-F*	ATGGTTCGAGAAAAAGTAAGA	1016	42
	*Pla-rpoA-R*	TTAAACCTTATTTTTCGGTAAATC		
3	*Pla-trnK-matK-F*	TTTAAGTCTATTTGAACCAAAGGTC	1016	44
	*Pla-trnK-matK-R*	GGGTTGCTAACTCAACGGTAG		
4	*Pla-trnK-rps16-F*	CAGGATCAGTCGTGGTCTTC	711	44
	*Pla-trnK-rps16-R*	TTATAGAATTGTTGCGATTGATGT

**Table 2:** Sequence characteristics of four intergenic spacer regions of *Platycodon grandiflorus* chloroplast genome.

Intergenic spacer regions	Sequence length (bp)	Sequence
*ndhC-trnV*	829	TAACTTTTTAGTTTAAGCAAGGATTGATTTTGCTCGAACCGTCTAGTTTACTTTGTTTACTCTAGGGCATGTCTTGTTTCAAGATTCATCAACGGAAATCCTATTTCCATTACATATATTTCCATTTGTTGTTATAGTTAGTATAACTAACTATTGCTCTTATCCAAATTTTCTTCTTTTTATCTCAGTCTCACCTAGGATTTTATCTAAAAAAGGGAATTCTAAAGAAAATTCTCATTTTTTGTTTAGAATTTATAATAATCAAATAAAGAGAAAATTTGAAATTCTTAGAAATTGATTGAAATTTAAATATAGTTTTCGTTTGCTTATTATTATTGTTATTATATAGATTTCACAATCTACTCGGGAGGTCTTTTTGTCTATTTTTCTTAGTGATTTACCATAGAGTAAGGAGTCATATGGAAGAGAATGGATAGAAATTTTCATCTCAAGATTTTGAATATTGGTGTGGGTATATTTATTTTAGTATTCGATTCTTCGTAGAATTATTAGAATCTAATTCATGGGGTAGGACTTTATATTCAAAAAATATGTAAAGAGAAGACTACCTTTGCTAGGTTTAGGTACAGGGATCCGACGAAAATCCTAGTTTTTTTATTTACAATGTTTAGAATGAAAGTTATCAAAGATATTCATCTTTCAAAGTTTAGCTTGTACACAAATACAGCCGGTCATTACTATACCCACAGTACTATTAGATTCGATTGGGGTTAGGTTAACTTGGAGTTTTTAAAGGGACTCGTGTTATAATTTTGACTTCCAAAATTCACTAGTATTTTTGCATTTATAGAGCTCT
*rpoA*	993	TAGAGTATTTACTCGGACACTGTGGAAGTGTTTGGAATCAAGAGCAGACAGTAAACGTCTTTATTATGGACGCTTTATTCTGTCTCCACTTATGAAAGGTCAAGCGGACACAATAGGCATTGCGATGCGACGAGCTTTACTTGGAGAAATAGAAGGGACATGTATCACACGCGCAAAATCTGAGAAAATACCACACGAATATTCTACCATAGTTGGTATTCAAGAATCAGTACATGAAATTTTATTGAATTTGCAAGAAATTGTAGTGAGAAGTCATCTATATGGAACTTGTGAGGCAGCCGTTTGTGCTAAGGGCCCCGGATATGTAACCGCTCAAGATATCATCGCGCCGCATTTTCTCAAAATCATTGATAATACACAGCATATAGCTACCTTGACAGAACCAATTGACTTGTGTATTTTTTTACAAATCGAAAGGAGTCATGGATGTGGATATCGTACAACAGAAAAGAGGATTATAGATGGAAGTTATCCTATAGGGGATGTATTAATGCCTGTTCGAAATGTGAATCATAGTATTCATTYTTATGGGACTGGAAATAAGAAACAAGAGATACTCTTTCTTGAAATCTGGACAAATGGAAGTTTGACTCCCAAAGAAGCACTTCGTGAAGCCACTCAGAATTTGATTGATTTGTTTATTCCCTTTTTATATACAGAAGAAGAAAACTTCCATTTAGAGGACAATCAACATATGGTTCCTYTACGCCCCTCTACCCTTCCTGATCCATTTGTCAAAATAACAAAAAACAAAAAAAAAATAGCATTGAAATCGATTTTTATTGACCAATCAGAATTACCACCCAGAGTCTATAATTGCCTTAAAAGTAATGATATAGATACATTATCAGACCTTTTGAATAACAGTCAAGAAGATCTTATGCAAATGGAACACTTTCGCGTAGAAGATGTCAAAAAGATAGTAGACATTCTCAAAAATAATTTCGGGATTGATTACG
*trnK-matK*	751	GACCAAAGGTCGAGGATTTTAGGGGTTATCAAATGATACATAGTGCAATCCAGTCAAAACAAGGTATTTTTTATAGTAAGAAAGGAATAGATACCTCGGATACAGGTAAACTTATCATCAGATTCTCTATCCTCTCGTTTTCCATTTAATTGAAGTAGTTTATATTCGTTCTAGGATAATTAAGATGTTTAGAAATCCTTTATTTTTTCAACCCAATCGCTCTTTTGATTTTGGAAATTTTTTGATCAATATACTGTTTCTTATACACATACATCTCCATTGTGGAATGGAGAATGATAATATTTAGGATTCATTAAATAATAAGGAATCCGCTCAGGGGAAAAGCCCTTCCCGCATCAGCCACTAATATATTTTTAACGTCTAATTAGATCGGGTAATCATTCAAATTAAGAATGGGAGCTCGTTACTTTTTCTTTCCTTATAATTTAAGGAAATTAATTGAAGCCACAGGGCTTTATCCATTTATTCATTCGACCCAACTTGAAATTGAATACATTTTGCTATCTTTCAATAAGGTAAAGAACGTTTTATACCGATCTGGCAAGAATAAAATATTCTCAGAACTCTTGATTGATACGACATGCTATTTTTTCCATTCATTCCCTTTCAGGATCAGTCGTGGTCTTCCAAACTTTACCGATGGTATGGATGAATCCATCACTTCATCCAAATGTGTAAAAGATCCTAGTCGCACAATAAAAGCCGAGTACTCTACCGTTG
*trnK-rps16*	690	TCTTCCAAACTTTACCGATGGTATGGATGAATCCATCACTTCATCCAAATGTGTAAAAGATCCTAGTCGCACTTAAAAGCCGAGTACTCTACCGTTGAGTTAGCAACCCGAATAAAATAAAAGAATGTAGATACAATCAGAATCAAAATAAATGATTAGACGAGGTAATCAAACCCTAAAAACACATTTTCTAATCGATTAGGAAAATAAAACGGAATAACTCAGACGAAAATACAATAAATTTGACGAGAAAAGAAAAAAACAAAAAATCTAAGATAAGGGATGAAAGCCCAAAAGGGGCCCATCATCCCTTATCTTATTCACTTTTTCAATATAAAATTATTCATTCAAAGCATAGACAAGGAACCTATTAGTTGAATAATGGAGTATATTTGTTCAATTCCTTGTTGTCAATATAAGGCAAACAATCAAATTTCAATTGAAATAAGATAAAAAAATTACTGAAGTATAAAAATATTTCGAATTGAAATTTGATGCGGGGACAATTACTTCAAAACTCCAAACTTCTTTAAAATCTGAAAAACCGTTTTTGTAGGTTTGGCACCCATTTCAAGAAAATATAGAATCGCGGATTTGTTTAAAGAAATTTGCTTGCTGCGCGGGTTATAAATTCCTACTTTCGCAAACTCGGCCCCATTTCGTCGAGATCGAACATCAAT

**Fig. 3:**
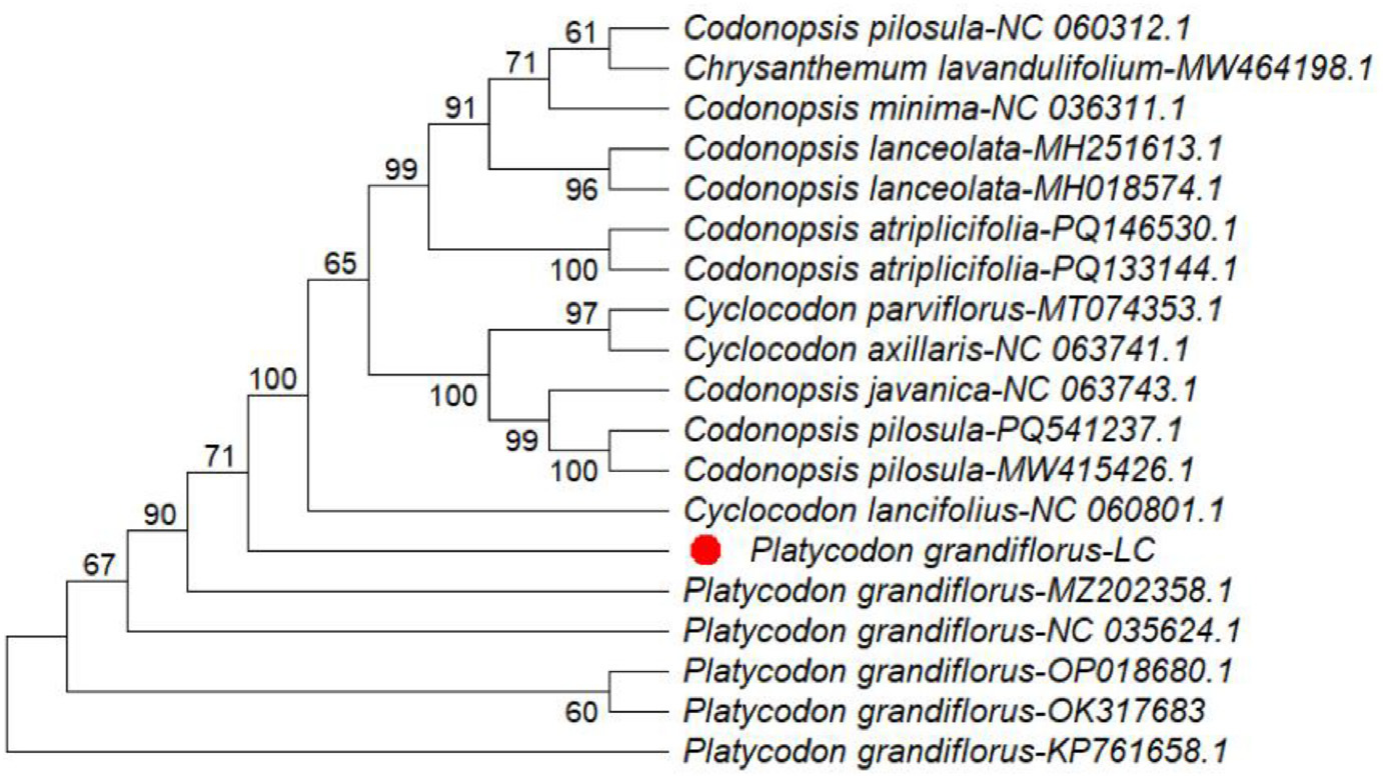
Phylogenetic tree construction using the *ndhC-trnV* intergenic spacer region of *Platycodon grandiflorus* chloroplast genome and of other similar species.

**Fig. 4:**
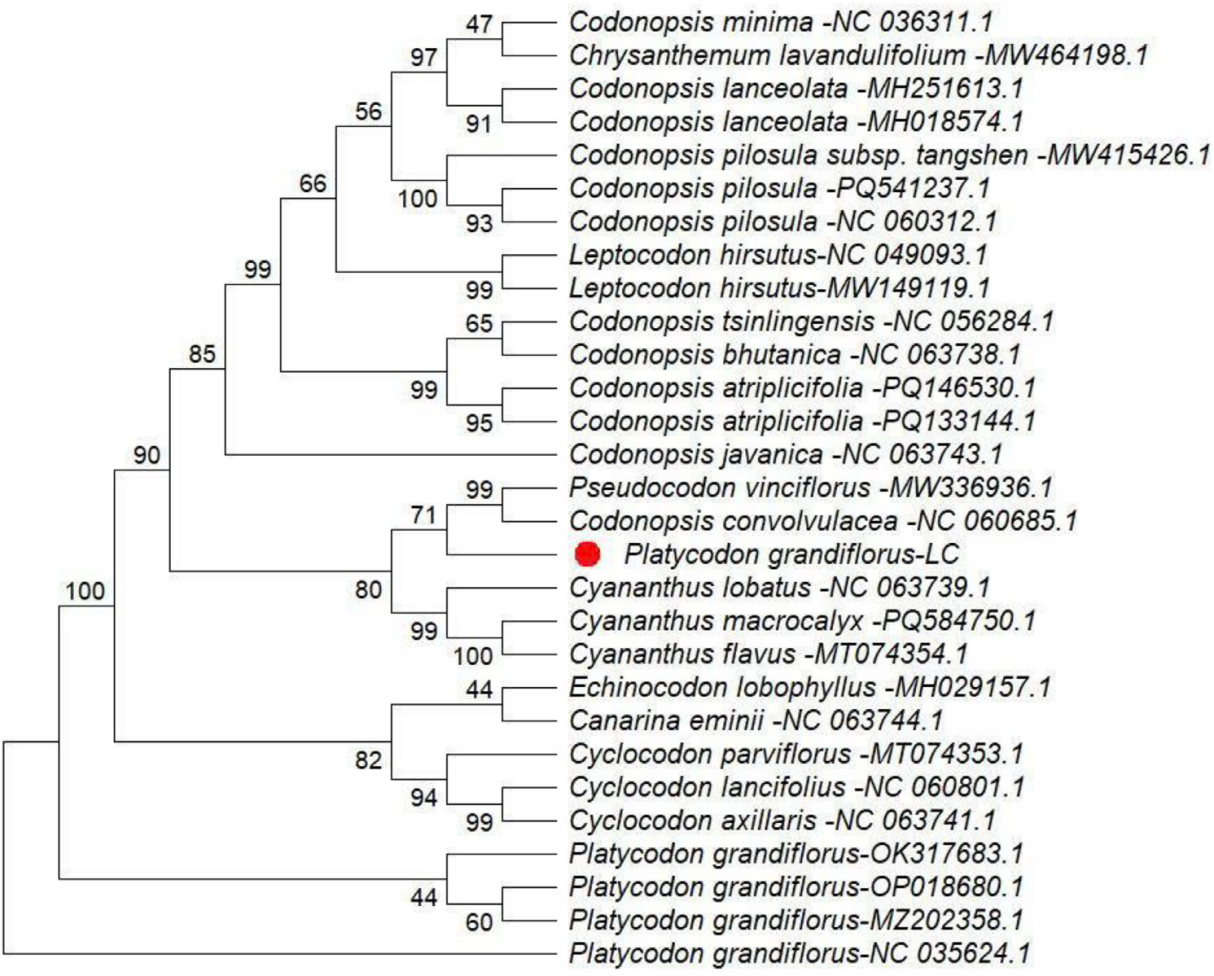
Phylogenetic tree construction using the *rpoA* gene of *Platycodon grandiflorus* chloroplast genome and of other similar species.

**Fig. 5:**
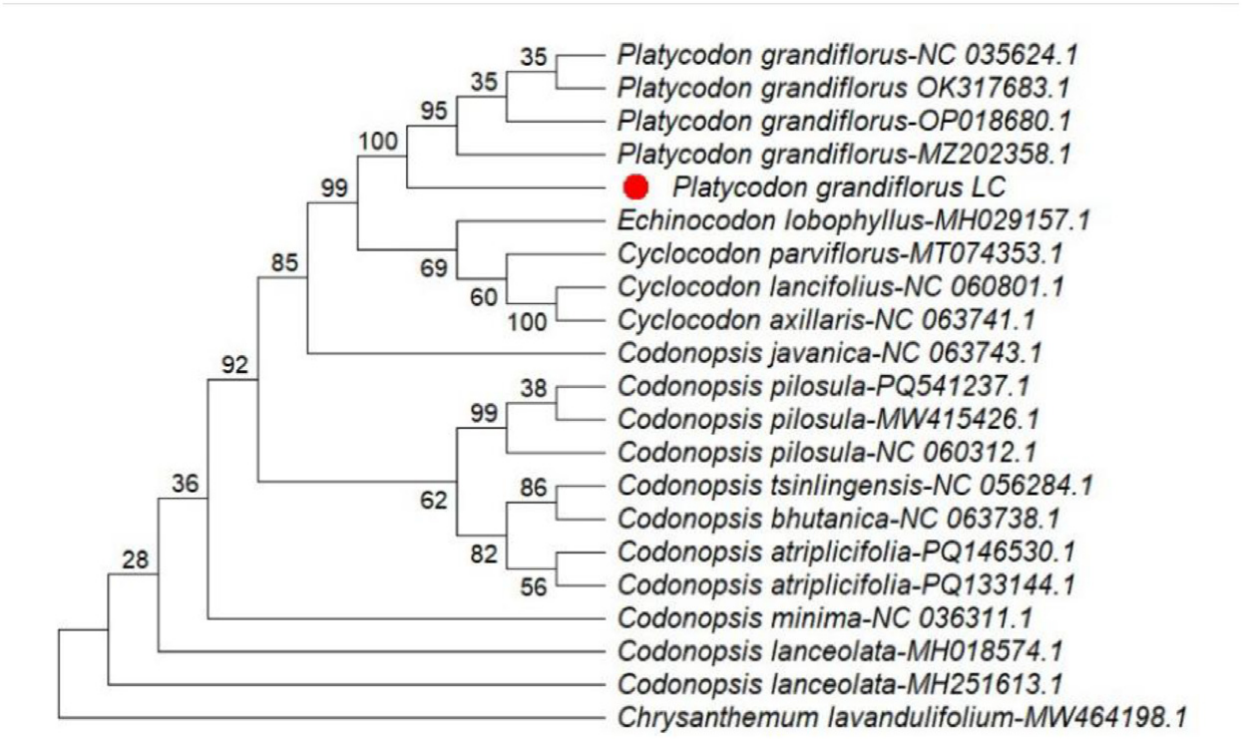
Phylogenetic tree construction using the *trnK-matK* intergenic spacer region of *Platycodon grandiflorus* chloroplast genome and of other similar species.

**Fig. 6:**
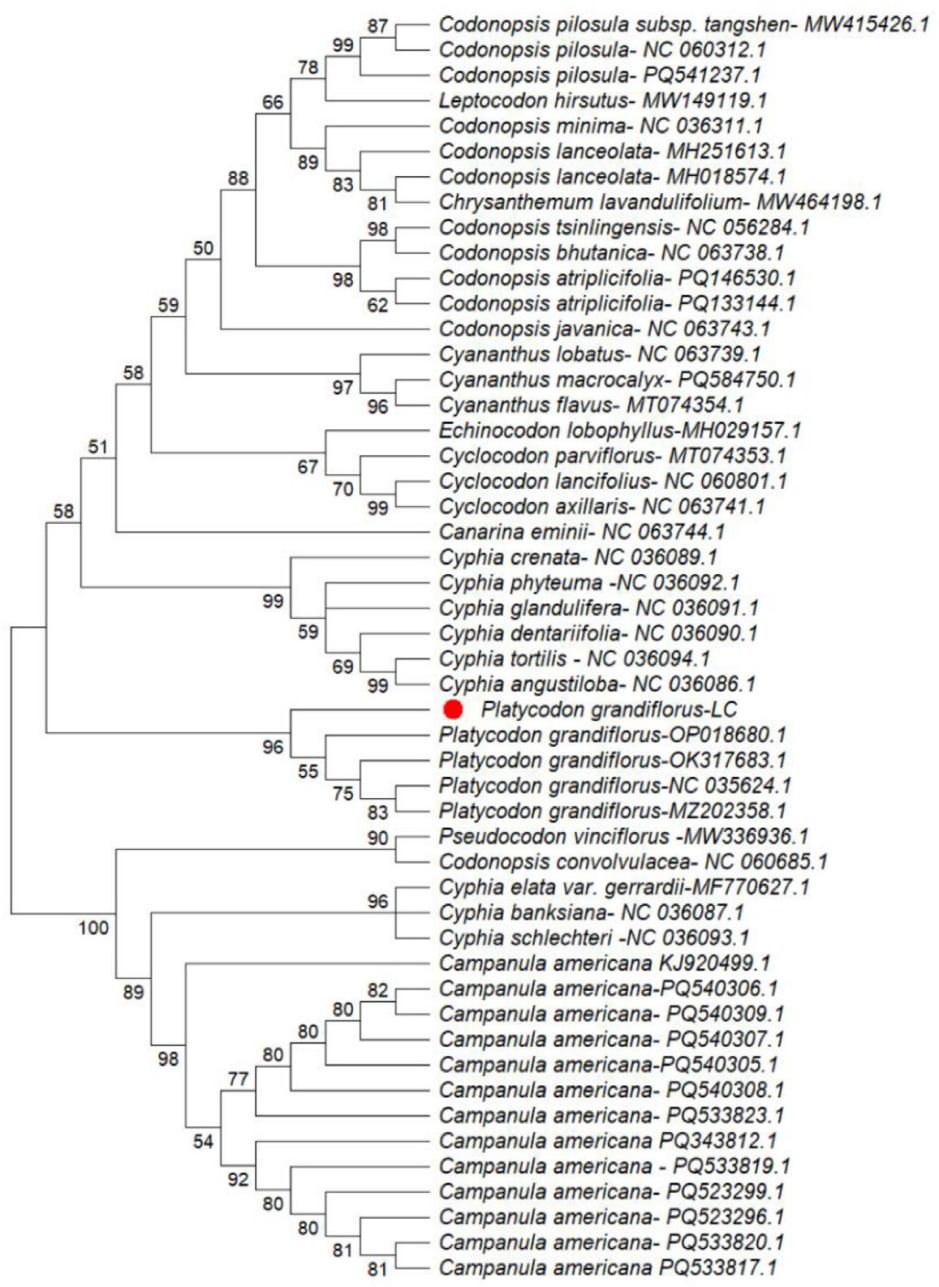
Phylogenetic tree construction using the *trnK-rps16* intergenic spacer region of *Platycodon grandiflorus* chloroplast genome and of other similar species.

Phylogenetic analyses were conducted using MEGA version 12.1 based on four chloroplast regions, including three intergenic spacers (*ndhC–trnV, trnK–matK, and trnK–rps16*) and the *rpoA* gene. The resulting phylogenetic trees indicated that *Platycodon grandiflorus* clusters within the family Campanulaceae and exhibits a close genetic relationship with related taxa, supported by high bootstrap values (96–100 %) (Fig. 3, Fig. 4, Fig. 5, Fig. 6). Among the analyzed regions, the three chloroplast intergenic spacers, *ndhC–trnV, trnK–matK, and trnK–rps16*, were identified as promising candidate DNA barcodes for reliable discrimination of *P. grandiflorus* from closely related species. Collectively, these results contribute to improving molecular identification, phylogenetic resolution, and quality control of *P. grandiflorus* and related taxa within Campanulaceae.

**Fig. 7:**
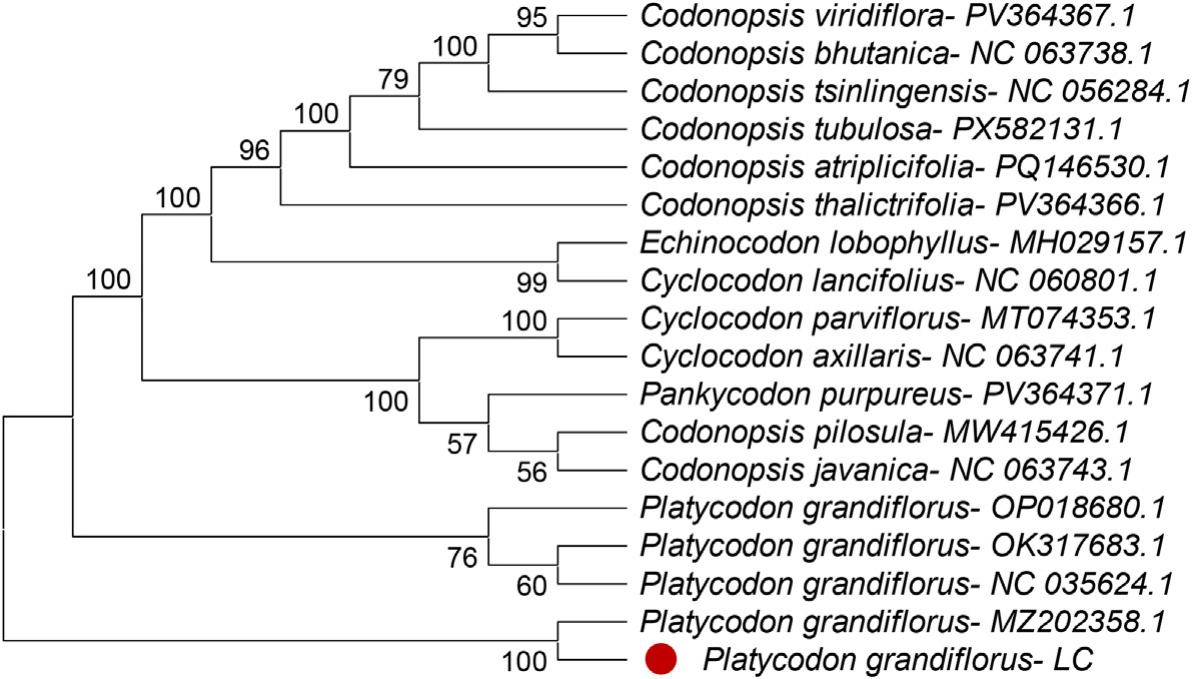
Phylogenetic tree construction using the *trnK-matK, trnK-rps16, ndhC-trnV, rpoA* of *Platycodon grandiflorus* chloroplast genome and of other similar species.

Phylogenetic reconstruction based on the combined dataset from four chloroplast regions (*trnK–matK, trnK–rps16, ndhC–trnV,* and *rpoA*) placed the *P. grandiflorus* LC sample in Clade 1, with strong bootstrap support (100 %) and accession MZ202858.1. This clade was clearly separated from the cluster containing the remaining P. grandiflorus samples, supported by a bootstrap value of 76 % (Fig. 7). These findings indicate detectable nucleotide-level divergence and demonstrate that multilocus chloroplast analyses substantially enhance phylogenetic resolution, thereby supporting their application in species-level identification.

## 4 Experimental Design, Materials and Methods

### 4.1 Sample collection and data source creation

Fresh samples of *Platycodon grandiflorus* (Jacq.) A.DC. (Voucher No. PLAG202507LC) were collected in July 2025 from Tan Chu Village, Lung Phinh Commune, Lao Cai Province, Vietnam (22°36′48.2″N, 104°17′12.0″E). Species identification was confirmed through comparative morphological assessment at the Department of Botany, Faculty of Biology, Thai Nguyen University of Education. Voucher specimens were deposited in the departmental herbarium. DNA sequences of related taxa within the Campanulaceae family were retrieved from the NCBI database using the BLAST search tool [5].

### 4.2 DNA extraction, PCR amplification, and sequencing

Total genomic DNA was isolated from fresh leaves using the cetyltrimethylammonium bromide (CTAB) method described by Shanghai-Maroof et al. [6]. DNA quality was verified by 0.8 % agarose gel electrophoresis, and purity was determined spectrophotometrically at 260 nm.

Four chloroplast regions, *ndhC–trnV, rpoA, trnK–matK*, and *trnK–rps16,* were amplified by PCR using primer pairs Pla-ndhCV-F/Pla-ndhCV-R, Pla-rpoA-F/Pla-rpoA-R, Pla-trnK-matK-F/Pla-trnK-matK-R, and Pla-trnK-rps16-F/Pla-trnK-rps16-R, designed from reference chloroplast sequences. Primer information and target fragment sizes are presented in Table 1.

Each PCR reaction (25 µL total volume) contained 12.5 µL of 2× master mix, 1.0 µL of each primer (10 µM), 2.0 µL of template DNA, and 8.5 µL of nuclease-free water. The thermal cycling profile consisted of an initial denaturation at 95 °C for 5 min, followed by 35 cycles of 95 °C for 30 s, 56 °C for 30 s, and 72 °C for 45 s, with a final extension at 72 °C for 5 min and storage at 4 °C for 10 min. PCR products were visualized on a 1.0 % agarose gel and purified using a Favorgen PCR cleanup kit (Taiwan).

Purified fragments were sequenced on an ABI 3130XL Genetic Analyzer (Applied Biosystems, USA) using the BigDye Terminator v3.1 kit. The obtained sequences were verified and aligned with reference data from GenBank using BLAST (NCBI) and BioEdit software [7].

### 4.3 PCR primer design

The complete chloroplast genome of *P. grandiflorus* (GenBank accession NC_035624.1) was retrieved from NCBI [3]. Nucleotide sequences corresponding to the *ndhC–trnV, rpoA, trnK–matK*, and *trnK–rps16* regions were extracted and used for primer design. Specific primer pairs were designed to amplify these target regions (Table 1).

### 4.4 Phylogenetic analysis

Phylogenetic relationships were inferred using MEGA version 12.1 [8], applying the Maximum Likelihood (ML) method under the Tamura–Nei substitution model [9]. The robustness of the inferred topology was assessed with 1000 bootstrap replicates, and a consensus tree was generated to illustrate evolutionary relationships among taxa [10]. Analyses were performed for four chloroplast regions: *ndhC–trnV* (19 sequences; 1058 aligned positions), *rpoA* (29 sequences; 1089 positions), *trnK–matK* (21 sequences; 820 positions), and *trnK–rps16* (50 sequences; 858 positions); and four chloroplast regions (*trnK–matK, trnK–rps16, ndhC–trnV,* and *rpoA*) from the collected sample were concatenated in the specified order, yielding a composite nucleotide sequence of 3219 bp. BLAST analysis of the concatenated sequence retrieved 17 closely related accessions from an initial dataset of 100 sequences, with total alignment scores ranging from 1432 to 1790, approximately 78 % query coverage, and nucleotide identity values of 93.16 % or higher.

## Limitations

None.

## Ethics Statement

The authors have read and followed the ethical requirements for publication in Data in Brief and confirmed that the current work does not involve human subjects, animal experiments, or any data collected from social media platforms.

## CRediT Author Statement

**Dung Manh Ngo:** Conceptualization, Investigation, Formal analysis, Visualization, Writing – original draft preparation; **Luong Trong Vu:** Formal analysis, Investigation; **Hung Duc Nguyen**: Sample collection, Formal analysis, Investigation; **Thuy Thi Thu Vu:** Conceptualization, Methodology, Data curation, Visualization, Data curation, Writing – original draft preparation; **Mau Hoang Chu**: Conceptualization, Methodology, Supervision, review and editing of the manuscript.

## Data Availability

Mendeley DataInternal Transcribed Spacer (ITS) and Several Chloroplast DNA Regions of Platycodon grandiflorus (Jacq.) A.DC. (Original data)
